# Assessment of Fatigue Life in Grouted Polyurethane Composites for Pavement Maintenance

**DOI:** 10.3390/ma18081806

**Published:** 2025-04-15

**Authors:** Fang Wang, Shiyi Zhang, Muyang Huang, Kai Liu, Chaoliang Fu

**Affiliations:** 1 School of Civil Engineering, Anhui Jianzhu University, Hefei 230601, China; fangwang@ahjzu.edu.cn (F.W.); zhangshiyi1201@stu.ahjzu.edu.cn (S.Z.); 2 School of Automobile and Traffic Engineering, Hefei University of Technology, Hefei 230009, China; 2023171696@mail.hfut.edu.cn (M.H.); liukai@hfut.edu.cn (K.L.); 3 Institute of Highway Engineering, RWTH Aachen University, 52074 Aachen, Germany

**Keywords:** polyurethane grouting, fatigue life, dissipated energy, pavement maintenance, prediction model

## Abstract

Polyurethane grouting technology is widely employed to maintain critical transportation infrastructure, including pavements, airports, and railways. After injection, foamed polyurethane bonds with surrounding aggregates to form a polyurethane–aggregate composite material (PACM). The gradation of aggregates in PACM, stress levels, and loading frequencies significantly influence fatigue performance under cyclic traffic loading. This study investigates the fatigue behavior of three distinct PACM gradation types through three-point bending fatigue tests under varying stress levels and loading frequencies. Results reveal that the finer gradations of PACM tend to exhibit higher flexural stiffness and longer fatigue life but also greater sensitivity to stress levels. Conversely, coarser gradations show lower stiffness but improved energy dissipation characteristics. Additionally, the flexural stiffness modulus, fatigue life, and cumulative dissipated energy decrease with increasing stress levels, while they grow with higher loading frequencies. In contrast, the dissipated angle follows an opposite trend. Additionally, mathematical models were developed to describe the evolution of dissipated energy, uncovering a three-stage pattern dominated by a prolonged plateau phase accounting for over 80% of the fatigue process. Based on this characteristic plateau, fatigue life prediction models were established for each gradation type, achieving high prediction accuracy with relative errors below 10%. These findings not only highlight the significant impact of aggregate gradation on PACM fatigue performance but also provide practical tools for optimizing material design in pavement maintenance.

## 1. Introduction

Road infrastructure is perpetually exposed to a range of environmental and mechanical challenges, including temperature variations, water-induced erosion, and the cyclic loading caused by growing traffic volumes [1,2,3,4]. The cumulative effects of these stressors often lead to the manifestation of common pavement distress, such as cracking, differential settlement, and surface depressions [5,6,7,8]. Repairing these issues in active roadways is not only time-sensitive but also technically demanding, necessitating innovative solutions that balance durability with efficiency. In this context, polyurethane grouting technology has emerged as a highly effective and versatile method for the rehabilitation of transportation infrastructure [9]. Its widespread adoption can be attributed to its ability to significantly reduce construction time while delivering repairs with exceptional strength and longevity [10,11,12]. The process begins with the injection of polyurethane grouting materials (PGMs) into damaged areas. Once injected, these materials undergo a multi-component polymerization reaction accompanied by rapid expansion. This expansion serves multiple functions: it fills voids, compacts loose aggregates, and establishes a robust bond with the surrounding materials. Importantly, the transformation of PGMs—from a liquid state to an expanding gel and ultimately to a solid form—ensures the creation of a durable repair solution capable of enduring the harsh conditions typical of modern roadways [13].

Currently, researchers have conducted a series of studies on PGMs. Gao et al. (2017) [14] performed cyclic compression loading tests to evaluate the compressive fatigue performance of PGMs. Their findings revealed a clear three-stage response pattern under repeated loading, with the fatigue failure stress threshold decreasing as material density increased. Similarly, Wei et al. (2017) [15] analyzed the fatigue behavior and failure mechanisms of PGMs across different densities, identifying the same three-stage process: elastic growth, steady development, and rapid failure. More recently, Liu et al. (2022) [16] examined the evolution of fatigue damage in PGMs from both experimental and theoretical perspectives. They demonstrated that fatigue life decreased with higher load magnitude and density but increased with faster loading speeds. Conversely, fatigue damage was exacerbated by higher load magnitudes and densities but mitigated by faster loading rates. Haruna et al. (2023) [17] studied the flexural response of concrete retrofitted with PGMs using three-point bending tests, and they found that PGMs significantly improved deflection, with top-surface retrofits showing linear deformation and bottom-surface retrofits exhibiting two deformation stages. Zhou et al. (2024) [18] evaluated the anti-cracking performance of polyurethane grout using the overlay test, demonstrating its superior durability and resistance to crack propagation at 25 °C compared to SBS-modified asphalt. Xiong et al. (2024) [9] explored using mineral powder in polyurethane grouting materials (PGMs) for asphalt crack repair. Excessive mineral powder reduced mechanical properties due to agglomeration, but its addition improved post-UV aging tensile strength by 12%.

In addition, in practical applications, PGMs are injected into pavements and interact with surrounding materials, forming composite structures. Therefore, researchers have thus begun to investigate the mechanical performance of PGMs when combined with pavement materials, aiming to understand their performance in actual engineering scenarios better. Opland and Barnhart (1995) [19] conducted tests on pavement stabilized with PGMs before and after repair. The results showed that after stabilization, improvements were observed in ride quality and load transfer efficiency near cracks (especially severe cracks) and joints. Crawley et al. (1996) [20] similarly found an increase in load transfer efficiency at joints after pavement stabilization. Additionally, they noted that in some cases, the PGM injection process created new voids beneath the panels, but reinjection helped mitigate this issue. Vennapusa and White (2015) [10] tested and analyzed deteriorated pavements that were repaired with PGMs. They found significant improvements in faulting and load transfer efficiency at cracks after the grouting repair. Cui et al. (2021) [11] investigated the fatigue performance of concrete–polyurethane composite materials under cyclic compressive loading. They concluded that the strain response of the composite materials can be divided into three stages: an initial stage with little change in cumulative strain, a steady stage where strain increases gradually, and a rapid stage where strain grows sharply, indicating fatigue failure. Among the studied factors, grout thickness had the most significant impact on fatigue performance, followed by stress ratio, void shape, and grout density. Zhong et al. (2023) [21] studied the bonding behavior between polyurethane and pavement materials. They varied interface roughness and polyurethane density, finding that polyurethane forms a dense film at the interface, with thickness increasing as density rises. Moreover, the interface roughness and polyurethane density significantly affect bonding performance, while stress–strain curves revealed key factors influencing failure mechanisms. Ran et al. (2023) [22] investigated the grouting mechanisms of polyurethane composites in asphalt pavement subsidence. CT analysis revealed that polyurethane foam filled the geopolymer, forming a stable structure. Ground-penetrating radar results showed no significant fluctuations or dislocations after grouting, indicating a strong grouting effect.

In summary, the application of PGMs for pavement repair has demonstrated feasibility (Table 1). However, when PGMs react with aggregates in infrastructure, they form a unique polyurethane–aggregate composite material (PACM). The mechanical behavior of this composite differs significantly from that of pure polyurethane and traditional concrete [23,24]. Although numerous studies have investigated the fatigue performance of mixed polyurethane concrete and inorganic polymer concrete [25,26,27,28,29], the fatigue characteristics of PACM—a distinct type of polyurethane concrete formed via grouting—are heavily influenced by the preparation process. As a result, PACM exhibits notable differences from these other materials. Moreover, current research on the fatigue behavior of such composites remains limited, hindering their broader application in engineering practices. Therefore, a comprehensive investigation into the fatigue performance of PACM is essential for enhancing the durability and extending the service life of transportation infrastructure.

**Figure 1:**
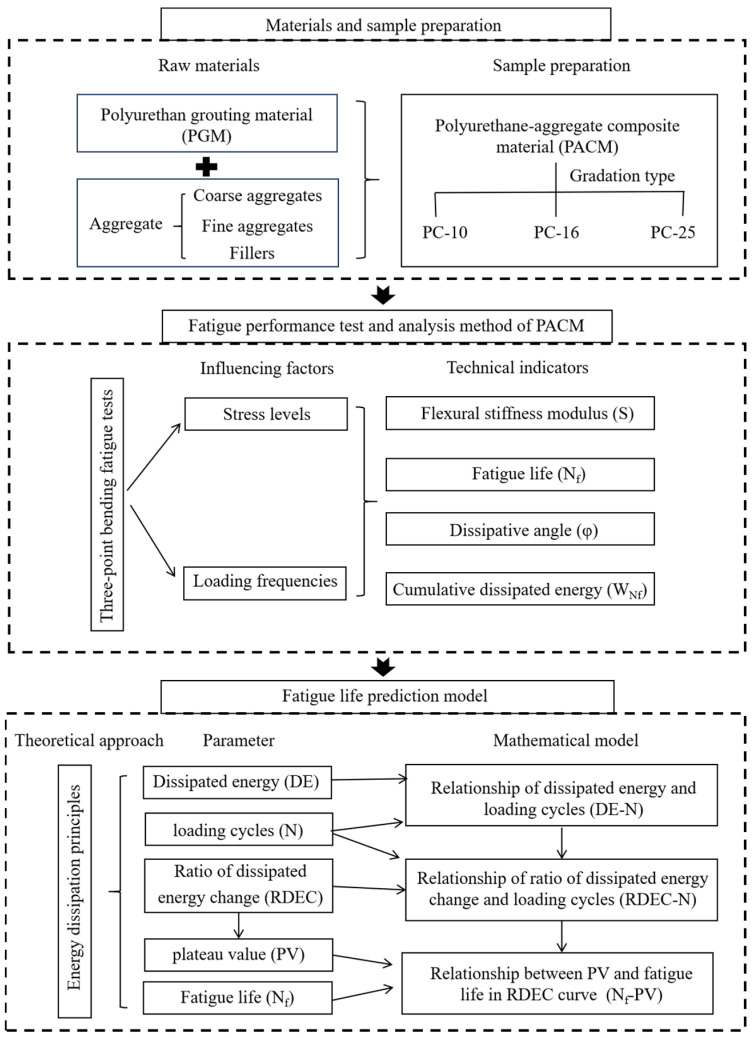
Technology roadmap.

To address this need, an innovative experimental and analytical framework for evaluating the fatigue behavior of PACM with three different gradation types under varying stress levels and loading frequencies aims to reflect realistic pavement conditions. This study is organized as follows: The next section details the material selection, gradation design, and specimen preparation of PACM. Section 3 describes the fatigue performance test and analysis method of PACM in detail and tests the bending stiffness modulus, fatigue life, dissipation angle, cumulative dissipated energy, and other indicators of PACM with different gradations. Compared with existing studies, the above experimental scheme takes into account the role of aggregate gradation in fatigue performance and can more realistically reflect the pavement conditions. In Section 4, based on the energy dissipation theory, an energy-based mathematical model is developed to characterize the dissipation behavior and energy damage evolution, and a fatigue life prediction equation is established through the power law relationship between dissipated energy and fatigue life, providing a new method to integrate energy mechanics into PACM fatigue analysis. These efforts not only contribute new insights into the fatigue mechanisms of polyurethane composite systems but also provide a methodological reference for the design and evaluation of advanced grouting materials in pavement engineering. The technical route of this article is shown in Figure 1.

## 2. Materials and Sample Preparation

### 2.1. Raw Materials

#### 2.1.1. Polyurethan Grouting Material

The grouting material used in this study is a two-component, non-water-reactive rigid polyurethane foam provided by Wanhua Chemical Group (Yantai, Shandong, China), formed through a chemical foaming reaction by mixing isocyanate and polyols in a 1:1 volume ratio. The reaction is facilitated by the addition of catalysts, blowing agents, and other additives. The isocyanate component is a black liquid with a density of 1.23 g/cm^3^ at room temperature, a -NCO content of 30.1 ± 0.5%, and a viscosity of 200 ± 50 mPa·s at 25 °C. The polyol component is a white liquid with a density of 1.02 g/cm^3^ at room temperature, a functionality of 2–3, and a viscosity of 730 ± 50 mPa·s at 25 °C.

#### 2.1.2. Aggregate

The coarse and fine aggregates used in this study are basalt sourced from Fuding City, Fujian Province. Basalt particles larger than 4.75 mm were classified as coarse aggregates, while those ranging between 0 and 4.75 mm were used as fine aggregates. The mineral filler was obtained by grinding limestone with particle sizes smaller than 0.075 mm. All their relevant technical indicators meet the requirements of the Technical Specifications for Construction of Highway Asphalt Pavements (JTG F40-2004) [30]. The technical properties of the coarse aggregates, fine aggregates, and fillers are shown in Table 2.

### 2.2. Sample Preparation

**Figure 2:**
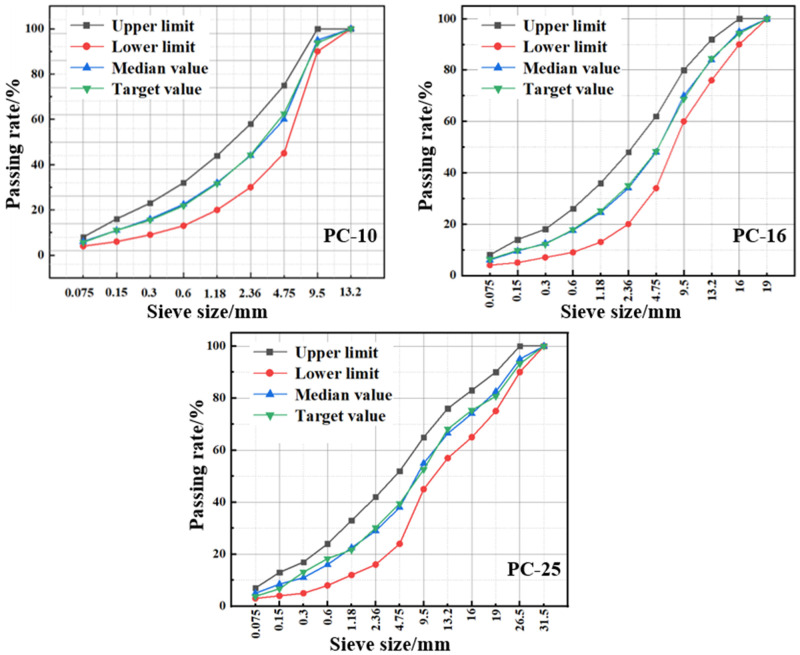
Three gradation curves of polyurethane–aggregate composite material.

**Figure 3:**
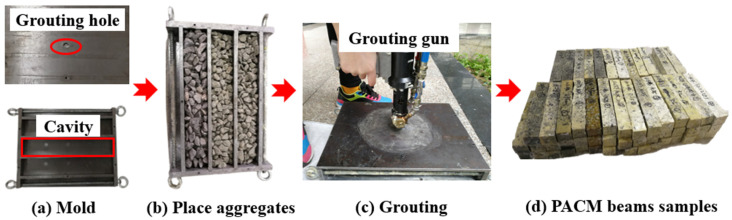
Preparation process of PACM beams.

To comprehensively consider the fatigue performance of the polyurethane–aggregate composite material (PACM) formed by combining polyurethane grouting materials with aggregates of different gradations, this study designed PACM gradations based on the aggregate gradations of AC-10, AC-16, and AC-25 asphalt mixtures specified in JTG F40-2004. It designated them as PC-10, PC-16, and PC-25, respectively. The gradation curves for the three mixtures are shown in Figure 2. To simulate the engineering environment of polymer grouting more accurately, a custom-designed grouting mold was independently developed, as shown in Figure 3a. The preparation involves injecting polyurethane grout into a sealed mold packed with aggregates, allowing the material to fully foam and expand, followed by demolding to obtain the final specimen. The specimens were utilized in subsequent three-point bending fatigue tests to thoroughly evaluate the fatigue performance of the PACM under traffic loading conditions.

As illustrated in Figure 3a, the custom-designed mold is a metal structure consisting of three interconnected rectangular cavities, each with dimensions of 300 × 50 × 50 mm, corresponding to the final dimensions of the PACM beam specimens. This design allows for the simultaneous production of three specimens with uniform density through a single injection process. The cavities are separated by two partitions, each equipped with a centrally located communication hole (diameter: 16 mm) to ensure uniform diffusion and expansion of the grouting liquid throughout the mold. To facilitate the grouting process, a small circular hole with a radius of 6 mm is positioned at the top of the upper cover plate, serving as the injection point for the polyurethane grout. The polyurethane content is maintained at 16% of the total mass of each specimen. Prior to molding, a thin layer of petroleum jelly was evenly applied to the inner walls of the mold to prevent adhesion and facilitate demolding. The walls were then covered with plastic wrap for additional protection. According to the gradation curve shown in Figure 1, aggregates and fillers were evenly distributed into each rectangular cavity of the mold. After sufficient vibration to ensure proper compaction and uniform filling of the aggregates, the mold was sealed using the upper cover plate, as depicted in Figure 3b. The grouting material was then injected into the mold using a grouting gun, as shown in Figure 3c. Following injection, the grouting hole was sealed with a plug to allow the polyurethane grout to fully react, foam, expand, and cure within the mold. After one hour, the mold was dismantled to retrieve the specimens, which were subsequently cured under dry and ventilated conditions for an additional 72 h, as shown in Figure 3d.

## 3. Three-Point Bending Fatigue Tests

Considering that PACM is subjected to both tensile and compressive stresses, this study adopts the three-point bending fatigue test to evaluate the fatigue performance of PACM. As a preliminary and foundational task, the static three-point bending test is crucial for determining the stress levels for subsequent three-point bending fatigue tests. The stress level refers to the ratio of the cyclic load applied during the fatigue test to the failure load observed in the monotonic failure test, with typical ratios ranging from 0.1 to 0.9. Based on the results of preliminary experiments (Table 3), the failure loads for different types of PACM were determined as 3.96 kN for PC-10, 2.67 kN for PC-16, and 3.23 kN for PC-25. Accordingly, stress levels of 0.3, 0.5, and 0.7 were selected for the fatigue tests in this study, meaning that the applied cyclic loads ranged from approximately 0.80 kN to 2.77 kN, depending on the PACM type and stress level. Additionally, considering the influence of different driving speeds on the fatigue performance of PACM, various loading frequencies were chosen for the analysis. According to previous research [16], a loading frequency of 10 Hz in the fatigue test approximately corresponds to a driving speed of 60–65 km/h. Therefore, 2, 5, and 10 Hz loading frequencies were selected for this study’s fatigue tests. The fatigue tests were performed under controlled stress mode using a sinusoidal wave [30]. The PACM specimens were prepared in a prismatic shape with dimensions of 300 mm (length) × 50 mm (width) × 50 mm (height).

**Figure 4:**
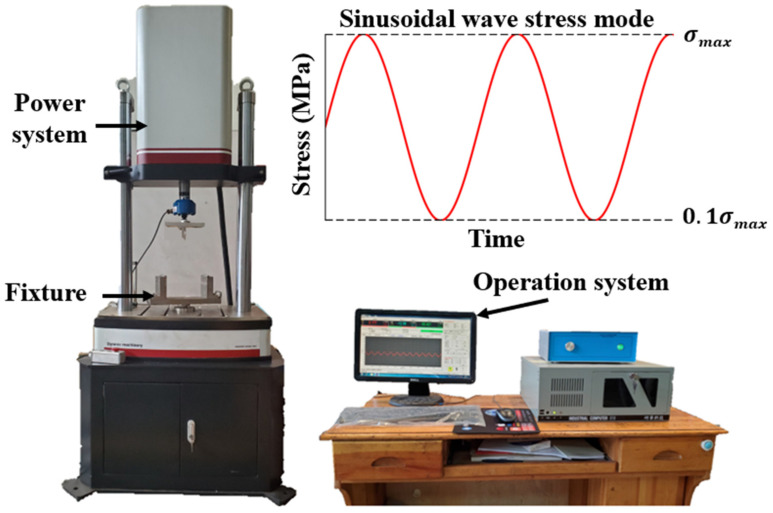
The three-point bending fatigue test platform.

Following the fatigue test protocols for asphalt mixtures specified in the Standard Test Methods of Bitumen and Bituminous Mixtures for Highway Engineering (JTG E20-2011) [31], three-point bending fatigue tests were conducted on PACM using a universal testing machine. The test was conducted in a temperature-controlled laboratory room, where the ambient temperature was maintained at 15 ± 1 °C using a calibrated air conditioning system. A temperature sensor was placed near the specimen to monitor and ensure consistent temperature during the test. During the fatigue tests, the mid-span vertical deflection of the specimen was recorded in real time using a linear variable displacement transducer (LVDT) with a precision of 0.01 mm, positioned directly under the loading point. The tensile strain was then calculated based on this displacement. The fatigue test was deemed complete when the specimen’s stiffness modulus decreased to 50% of its initial value, with the corresponding number of loading cycles defined as the material’s fatigue life. The test setup consisted of one upper indenter and two lower supports, each constructed from fixed steel bars with a radius of 10 mm. The distance between the two lower supports was set at 200 mm, with the upper indenter positioned centrally. To ensure reliability, three parallel samples were tested for each stress level. The three-point bending fatigue testing apparatus, supplied by Tianjin Gangyuan Testing Instrument Co., Ltd. (Tianjin, China), primarily comprises a power system and an operation control system, as illustrated in Figure 4.

After completing the tests, the flexural stiffness modulus (*S*) of PACM with different gradations can be calculated by Equations (1)–(3).

(1) $$S=\frac{\sigma }{\varepsilon }$$

(2) $$\sigma =\frac{3PL}{2bh^{2}}$$

(3) $$\varepsilon =\frac{6hd}{L^{2}}$$

where *S* is the flexural stiffness modulus (N/mm^2^); *σ* is the maximum bending stress (N/mm^2^); *ε* is the maximum flexural tensile strain (dimensionless); *P* is the failure load when the sample failure (N); *L* is the distance between support rollers (mm); *b* is the width of the sample (mm); *h* is the height of the sample (mm); *d* is the maximum displacement in the center of the sample (mm).

Additionally, evaluation indices based on energy dissipation theory were used to investigate the effects of loading frequency and stress level on the fatigue performance of PACM with different gradations, including the dissipative angle φ and cumulative dissipated energy $W_{Nf}$. The dissipative angle φ refers to the phase difference angle between stress and strain. In viscoelastic materials, there is usually a time delay between the applied stress and the resulting strain. This delay can be expressed as an angle, known as the dissipative angle, and can be calculated using Equation (4). Cumulative dissipated energy, $W_{Nf}$, refers to the total amount of energy dissipated by the material in each loading cycle throughout the entire fatigue test. It is an important indicator of energy loss in the material under repeated loading and can be calculated using Equations (5) and (6).

(4) $$\varphi \left(N\right)=\omega ∆t\left(N\right)=2\pi f∆t(N)$$

(5) $$W_{Nf}=\sum_{i=1}^{Nf}∆W_{i}$$

(6) $$∆W_{N}=\pi \sigma_{t}(N)\varepsilon_{t}(N)\sin\;[\phi (N)]$$

where ω is the loading angular frequency; $∆t\left(N\right)$ is the time of the strain peak lagging behind the peak stress when the loading number is *N*; $∆W_{N}$ is the dissipated energy when the loading number is *N*.

## 4. Establishment of Fatigue Life Prediction Model

To establish a robust fatigue life prediction model for PACM, this study adopts a systematic theoretical approach based on energy dissipation principles. The modeling framework is divided into three key stages: (1) the relationship between dissipated energy (*DE*) and loading cycles, (2) the introduction of the ratio of dissipated energy change (*RDEC*) to capture dynamic fatigue behavior, and (3) the development of a fatigue life prediction model using the plateau value (*PV*) of the RDEC curve. Below, we describe each stage in detail.

### 4.1. Relationship of Dissipated Energy and Loading Cycles

Fatigue damage in materials can be quantified by the energy dissipated during cyclic loading [32]. In this study, the relationship between *DE* and the number of loading cycles (*N*) is modeled using a power-law relationship. This relationship assumes that *DE* varies nonlinearly with *N* due to the interplay of viscoelastic deformation and irreversible damage. Specifically, the total dissipated energy during each cycle is decomposed into two components: energy contributing to irreversible damage deformation ${DE}_{N}^{\delta }$ and energy consumed by viscoelastic deformation ${DE}_{N}^{\psi }$, as expressed in Equation (7):

(7) $${DE}_{N}={DE}_{N}^{\delta }+{DE}_{N}^{\psi }$$

where ${DE}_{N}$ is the dissipated energy at the *N*th loading cycle; ${DE}_{N}^{\delta }$ is the dissipated energy causing material damage at the *N*th loading cycle; ${DE}_{N}^{\psi }$ is the dissipated energy due to delayed viscoelastic deformation at the *N*th loading cycle.

Based on prior studies [33], the variation of *DE* with *N* can be described using a power-law function, as shown in Equation (8):

(8) $$DE(N)=1/(a+bN^{c}+dN^{e})$$

where *N* is the number of loading cycles; *a*, *b*, *c*, *d*, and *e* are the parameters. This mathematical model captures the characteristic three-stage evolution of *DE*: an initial sharp decrease, followed by a prolonged plateau phase, and finally a rapid increase leading to failure.

### 4.2. Relationship of Ratio of Dissipated Energy Change and Loading Cycles

While the *DE*-*N* relationship provides valuable insights into fatigue behavior, it does not fully account for the dynamic and nonlinear characteristics of energy dissipation during cyclic loading. To address this limitation, the *RDEC* is introduced as a more effective metric for analyzing fatigue progression [34]. *RDEC* measures the relative change in dissipated energy between consecutive loading cycles, as defined in Equation (9):

(9) $$RDEC=\frac{\left|{DE}_{N+1}-{DE}_{N}\right|}{{DE}_{N}}$$

By substituting the *DE*-*N* model (Equation (8)) into Equation (9), the mathematical relationship for *RDEC* is derived as follows:

(10) $$RDEC=\frac{\left|{DE}_{N+1}-{DE}_{N}\right|}{{DE}_{N}}=\left|\frac{a+bN^{c}+dN^{e}}{a+b({N+1)}^{c}+d{\left(N+1\right)}^{e}}-1\right|$$

This equation allows for the characterization of the *RDEC*-*N* curve, which typically exhibits three distinct stages: an initial rapid decrease, a stable plateau phase, and a final sharp increase. The *PV* during the second stage serves as a critical parameter for evaluating fatigue behavior, as it reflects a balance between viscoelastic deformation and irreversible damage.

### 4.3. Establishment of Prediction Model

Several fatigue life prediction models have been proposed in the literature, including stress-based (*S–N*) models, strain-based models, and energy-based models. Stress-based models typically describe empirical relationships between stress amplitude and fatigue life but often fail to capture the complex damage evolution in viscoelastic or polymer-based materials. Strain-based models offer improved accuracy for ductile materials and can account for localized deformations; however, they rely heavily on precise strain measurements, which are difficult to obtain in composite or grouted systems. In contrast, energy-based models consider the energy dissipated during cyclic loading as a damage-driving parameter, making them particularly suitable for materials exhibiting time-dependent or nonlinear behavior. In addition, the *PV* observed in the *RDEC* curve is closely linked to the fatigue life of the material. This stable phase dominates the fatigue process, accounting for over 80% of the total duration. Based on this observation, a fatigue life prediction model is developed, linking the *PV* to the fatigue life ($N_{f}$) through a power-law relationship [35], as shown in Equation (11):

(11) $$N_{f}={A(PV)}^{B}$$

where $N_{f}$ is the fatigue life; *A* and *B* are the parameters. This model enables accurate and comprehensive predictions of fatigue life by incorporating the dynamic characteristics of energy dissipation during the fatigue process.

## 5. Results and Discussion

### 5.1. Flexural Stiffness Modulus Analysis

**Figure 5:**
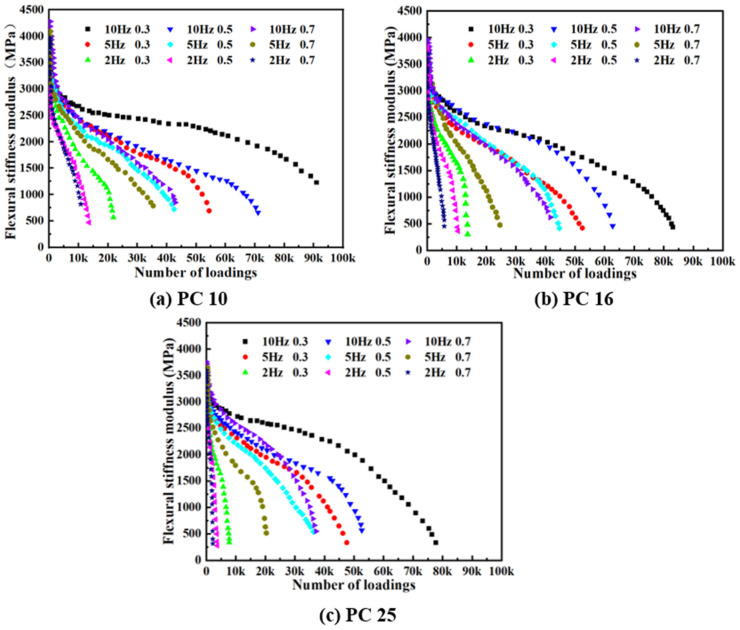
Relationship between flexural stiffness modulus and number of loadings of PACM with different gradation under different stress levels and loading frequencies.

The relationship between the flexural stiffness modulus (*S*) and the number of load cycles for different gradation types of PACM under varying stress levels and loading frequencies is depicted in Figure 5. The results clearly indicate that *S* decreases as the number of loading cycles increases. The attenuation pattern of *S* under different test conditions can be categorized into three distinct phases. In the first phase, *S* rapidly declines as the number of loading cycles increases, indicating a high degradation rate. Wei et al. (2017) [15], who also studied the fatigue behavior of PACM, found that this initial stage can be attributed to the inherent microstructural characteristics of PACM, a composite material with microcracks, voids, and weak interface regions. These defects act as stress concentration points under cyclic loading, causing the material properties to deteriorate rapidly. In the second phase, as loading continues, *S* stabilizes, and the rate of reduction slows down, following a nearly linear downward trend. This phase represents the most prolonged period of stiffness evolution, where the material experiences a more gradual and consistent change. The gradual decline in *S* during this phase reflects the material’s adjustment to cyclic loading, as the internal microstructure undergoes less severe changes compared to the initial phase. In the third phase, *S* declines sharply once again as the number of load cycles increases, ultimately leading to fatigue failure. This final phase is characterized by the rapid accumulation of damage, which overwhelms the remaining structural integrity of the material, resulting in a steep reduction in stiffness and eventual failure. The above discussion is in good agreement with the findings of Cui et al. (2021) [11]. This progression in *S* reduction across the three phases is indicative of the material’s fatigue behavior under cyclic loading, where early rapid damage is followed by a period of relative stability before a final, catastrophic failure occurs.

Additionally, it is observed that the initial *S* is highest for PC-10 PACM, followed by PC-16 PACM, and then PC-25 PACM. From the overall trend of the *S* attenuation curves, a clear pattern emerges: within the same gradation of PACM, the higher the applied stress level at a given loading frequency, the steeper the slope of the curve. This suggests that the higher the stress level endured by the sample, the more pronounced the attenuation in *S* becomes. Similarly, when comparing the *S* attenuation behavior at loading frequencies of 2, 5, and 10 Hz, it is evident that the curves for different gradation types of PACM at 2 Hz exhibit a steeper decline compared to those at 5 Hz and 10 Hz. This indicates that lower loading frequencies lead to faster *S* reduction across all gradation types, resulting in smaller residual *S* values. This is because the lower frequency likely allows for more prolonged loading per cycle, which exacerbates the material’s fatigue, leading to accelerated stiffness loss.

### 5.2. Fatigue Life Analysis

**Figure 6:**
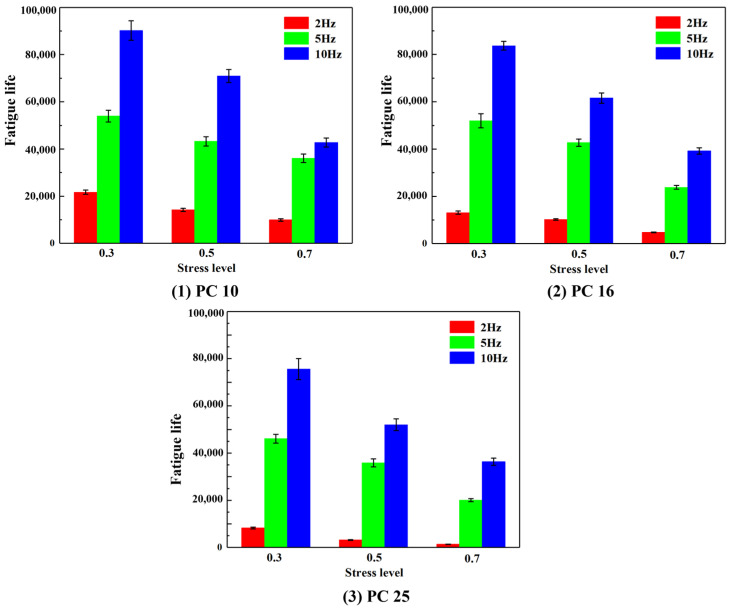
Fatigue life of PACM with different gradation under different stress levels and loading frequencies.

Fatigue life refers to the number of loading cycles that PACM can endure under repeated cyclic stress until the specimen ultimately fails. The fatigue life of different gradation types of PACM under varying stress levels and loading frequencies is presented in Figure 6. The results show a consistent trend: regardless of gradation, fatigue life decreases as the stress level increases, demonstrating that higher stress levels negatively impact the fatigue performance of PACM. For instance, in PC-10 PACM at loading frequencies of 2 Hz, 5 Hz, and 10 Hz, when the stress level increases from 0.3 to 0.5, the fatigue life decreases by 34.44%, 19.94%, and 21.34%, respectively. Similarly, when the stress level increases from 0.5 to 0.7, the fatigue life decreases by 29.83%, 16.55%, and 39.64%, respectively. These results confirm that fatigue life is highly sensitive to stress levels, with higher stress leading to accelerated fatigue damage. Additionally, at a constant stress level, the fatigue life of PACM increases with loading frequency, indicating that frequency significantly affects fatigue performance. For example, in PC-16 PACM at stress levels of 0.3, 0.5, and 0.7, increasing the loading frequency from 2 Hz to 5 Hz results in fatigue life increases of 2.95, 3.18, and 3.93 times, respectively. Further increasing the frequency from 5 Hz to 10 Hz leads to smaller gains in fatigue life, with increases of 0.61, 0.44, and 0.64 times, respectively. This suggests that while higher frequencies enhance fatigue resistance, the rate of improvement diminishes as frequency continues to rise, likely due to reduced crack propagation per cycle at higher frequencies. This view is also supported by the research of Wu et al. (2020) [36].

The gradation effect on fatigue life is also evident, with the general ranking among the three PACM types being PC-10 > PC-16 > PC-25. For instance, at a loading frequency of 10 Hz, when the stress level is 0.3, the fatigue life of PC-10 PACM is 7.74% and 19.25% higher than PC-16 and PC-25, respectively. At a stress level of 0.5, the fatigue life of PC-10 PACM is 13.14% and 36.28% higher than PC-16 and PC-25, respectively. When the stress level reaches 0.7, the fatigue life of PC-10 PACM remains 9.14% and 17.67% higher than PC-16 and PC-25, respectively. This trend confirms that aggregate size influences the fatigue performance of PACM. The smaller the aggregate size, the greater the contact area and interlocking force, which enhances fatigue resistance by limiting crack initiation and propagation. Therefore, the selection of aggregate gradation is a crucial factor in optimizing the fatigue performance of PACM, particularly under high-frequency cyclic loading conditions.

### 5.3. Dissipated Angle Analysis

The variations in the dissipated angle (φ) and its standard deviation for different gradation types of PACM are presented in Table 4. It is observed that φ increases with rising stress levels. For instance, at a loading frequency of 2 Hz, the dissipated angles for PC-16 PACM at stress levels of 0.3, 0.5, and 0.7 are 26.8°, 31.2°, and 37.5°, respectively. This increase in φ is attributed to the greater deformation of PACM under higher stress levels for the same number of load cycles, resulting in higher energy dissipation. At a constant stress level, the dissipated angle φ decreases as the loading frequency increases. For example, at a stress level of 0.3, increasing the loading frequency from 2 Hz to 10 Hz reduces the dissipated angles of PC-10, PC-16, and PC-25 by 2.9°, 3.3°, and 8.3°, respectively. This suggests that higher frequencies lead to a smaller viscous contribution and a more elastic response in the material. Under the same stress level and loading frequency, the ranking of φ is PC-10 < PC-16 < PC-25, indicating that the dissipated angle increases with larger aggregate sizes. A larger dissipated angle signifies a greater viscous response, meaning the material exhibits more viscous behavior relative to elastic behavior. As a result, more energy is dissipated in each loading cycle, which can reduce the material’s fatigue resistance. The magnitude of the dissipated angle under cyclic loading is closely related to the formation and propagation of fatigue cracks. If the elastic component of the material bears a larger proportion of the cyclic load during fatigue crack development, the φ at fatigue failure will be smaller. Conversely, if the viscous component dominates during crack propagation, the φ at fatigue failure will be larger [37]. This relationship underscores the importance of balancing viscous and elastic properties in optimizing the fatigue performance of PACM.

### 5.4. Cumulative Dissipated Energy Analysis

The cumulative dissipated energy ($W_{Nf}$) and its standard deviation of PACM with different gradation types are presented in Table 5. It is evident that $W_{Nf}$ decreases as the stress level increases. For instance, at a loading frequency of 2 Hz, the $W_{Nf}$ for PC-10 PACM at stress levels of 0.3, 0.5, and 0.7 are 68.388, 59.858, and 43.204 MJ/m^3^, respectively. In contrast, for PC-25 PACM, the corresponding $W_{Nf}$ are 25.361, 13.271, and 6.387 MJ/m^3^. This demonstrates that higher stress levels result in lower cumulative dissipated energy, which correlates with reduced fatigue life. The principle of dissipated energy suggests that materials with higher $W_{Nf}$ generally exhibit longer fatigue lives (Shen et al., 2006 [33]). When the stress level is held constant, the $W_{Nf}$ of PACM increases with the loading frequency. For example, at a stress level of 0.3, increasing the loading frequency from 2 Hz to 10 Hz results in $W_{Nf}$ increases of 170.46%, 346.11%, and 523.40% for PC-10, PC-16, and PC-25 PACM, respectively. This indicates that loading frequency significantly influences $W_{Nf}$. Dissipated energy refers to the energy consumed by the material when subjected to external forces. In other words, the greater the work done on the material by these external forces, the larger the $W_{Nf}$. Although each loading cycle at higher frequencies involves less energy dissipation per cycle, the total number of cycles increases with frequency, leading to higher overall $W_{Nf}$ values. At a constant stress level and loading frequency, the ranking of $W_{Nf}$ follows the order PC-10 > PC-16 > PC-25, which is consistent with previous analyses of fatigue life and dissipated angle. This trend reinforces the conclusion that smaller aggregate sizes enhance the energy dissipation capacity and improve fatigue resistance, as seen in PC-10 PACM.

### 5.5. Prediction Model of Fatigue Life

The fatigue life prediction model for PACM was established based on the *PV* of the ratio of *RDEC*, as described in Equation (11). The fitting parameters for different PACM gradations are presented in Table 6, with correlation coefficients (R^2^) exceeding 0.95 for all gradation types, indicating the high accuracy of the model. Specifically, the parameter A ranges from 0.0864 to 2.1717, while B varies between −1.515 and −1.072, reflecting the material-specific dependence of fatigue life on *PV*.

**Figure 7:**
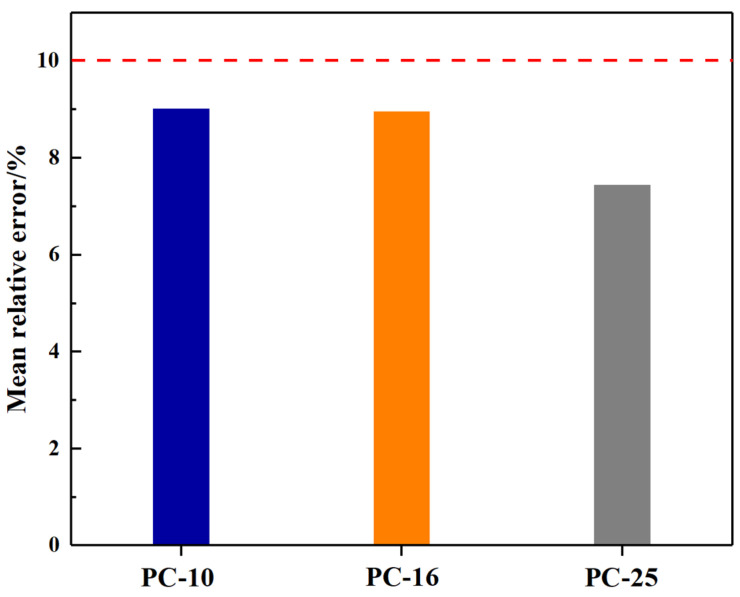
Mean relative error of PACM with different grading types.

In addition, model validation was performed using the mean relative error (MRE), calculated according to Equation (12).

(12) $$MRE=\frac{1}{n}\sum_{i=1}^{n}\frac{\left|{{PRE}_{i}-EXR}_{i}\right|}{{EXR}_{i}}$$

where *n* is the number of samples; ${PRE}_{i}$ is the predicted value; ${EXR}_{i}$ is the measured value. As shown in Figure 7, the *MRE* values for all gradation types are below 10%, confirming the model’s reliability and precision. Notably, the model demonstrates strong consistency across different gradation types, with R^2^ > 0.95 and MRE < 10%, underscoring its robustness. Furthermore, the *PV* emerges as a universal metric for fatigue life prediction, independent of material type or loading conditions. These results highlight the model’s effectiveness in predicting the fatigue performance of PACM under various scenarios.

## 6. Conclusions

In order to more accurately simulate the engineering environment of polymer grouting, this study first used polyurethane grouting technology to prepare three polyurethane aggregate composites (PACMs) with different gradations and evaluated the fatigue properties of PACMs under different gradations, stress levels, and loading frequencies through three-point bending fatigue tests. Secondly, by systematically analyzing the changes in key parameters such as bending stiffness modulus, fatigue life, dissipation angle, and accumulated dissipated energy, a mathematical model based on dissipated energy and dissipated energy ratio was established. Finally, a fatigue life prediction model for PACM under different gradation types was established based on the mathematical model. The main conclusions are as follows:

(1) The bending stiffness modulus (*S*) of PACM was determined by a three-point bending test as a function of stress level and loading frequency. The results showed that the *S* of PACM decreased with increasing stress level and increased with increasing loading frequency.
(2) By comparing the fatigue properties of PACM under different grading types, it was found that regardless of the grading type, its fatigue life will shorten with the increase in stress level and increase with the growth in loading frequency. Under all conditions, PC-10 has the most extended fatigue life; that is, the smaller the aggregate size, the stronger the fatigue resistance of PACM.
(3) The dissipation angle of PACM increases with the increase in stress level and decreases with the increase in loading frequency; under the same stress level and loading frequency, the dissipation angle of PACM with different gradations is ranked as PC-10 < PC-16 < PC-25.
(4) The cumulative dissipated energy of PACM decreased with the increase in stress level and increased with the increase in loading frequency. Under the same stress level and loading frequency, the order of cumulative dissipated energy of PACM with different gradings was PC-10 > PC-16 > PC-25.
(5) This study correlated the platform value (PV) of the ratio of dissipated energy change (*RDEC*) with the fatigue life of PACM and established a fatigue life prediction model for PACM of different grading types. Compared with the measured values, the theoretical values of the prediction model were in good agreement with the experimental values, with a relative error of less than 10%.

Although this study provides valuable insights into the fatigue behavior of PACMs, several aspects require further investigation to enhance the applicability of these findings. One crucial area for further research is long-term performance and environmental effects. The effects of environmental factors such as temperature variations, moisture, and aging on the fatigue behavior of PACMs need to be explored to simulate real-world conditions better. Understanding how these external influences affect material performance over time will be crucial for ensuring durability in practical applications. Additionally, developing advanced numerical models, such as finite element or discrete element simulations, would allow for in-depth analysis of stress distribution, crack propagation, and energy dissipation mechanisms. These simulations can help predict performance under different conditions and optimize material design before experimental validation.

## Figures and Tables

**Table 1:** Summary of previous studies on polyurethane grouting materials.

Literatures	Focus	Method	Key Findings
Gao et al. (2017) [14]	Compressive fatigue of PGM	Cyclic compression loading tests	Identified a three-stage fatigue response; failure stress threshold decreases with higher density
Wei et al. (2017) [15]	Fatigue behavior across densities	Experimental analysis	Confirmed three fatigue stages: elastic growth, steady development, and rapid failure
Liu et al. (2022) [16]	Fatigue damage evolution	Experimental and theoretical study	Fatigue life decreases with higher load and density, increases with faster loading rates
Haruna et al. (2023) [17]	Flexural response of retrofitted concrete	Three-point bending tests	PGMs improved deflection; top-surface retrofits showed linear deformation; bottom-surface had two stages
Zhou et al. (2024) [18]	Anti-cracking performance	Overlay Test	Polyurethane grout outperformed SBS asphalt in crack resistance at 25 °C
Xiong et al. (2024) [9]	Mineral powder in PGMs	Mechanical testing and UV aging	Excess powder reduced strength due to agglomeration; improved tensile strength by 12% post-aging
Opland & Barnhart (1995) [19]	Pavement stabilization using PGM	Field test before/after repair	Enhanced ride quality and load transfer at cracks and joints after PGM injection
Crawley et al. (1996) [20]	Joint performance post stabilization	Field evaluation	Increased load transfer efficiency; voids from initial injection mitigated by reinjection
Vennapusa & White (2015) [10]	Deteriorated pavement repair	Field tests	Faulting and load transfer improved after PGM grouting
Cui et al. (2021) [11]	Fatigue of concrete-PGM composites	Cyclic compressive loading	Identified three-stage strain response; grout thickness had most significant impact
Zhong et al. (2023) [21]	Bonding between PGM and pavement	Interface roughness & density tests	Denser polyurethane forms thicker film; roughness and density affect bond strength and failure modes
Ran et al. (2023) [22]	Grouting mechanism in asphalt subsidence	CT and GPR analysis	Polyurethane foam filled voids forming stable structures; no displacement after grouting

**Table 2:** Technical properties of coarse aggregates, fine aggregates, and fillers.

Type		Results	Specification
Coarse aggregate	Density [g/cm^3^]	2.73	/
	Crushing value [%]	21.7	≤26
	Los Angeles abrasion value	25.2	≤28
	Water Absorption [%]	1.6	≤2.0
	Content of needle and flaky particles [%]	11.36	≤15
	Content of soft particles	1.2	≤3
	Soundness	10.5	≤12
Fine aggregate	Density [g/cm^3^]	2.687	/
	Sand equivalent [%]	67	≥60
	Mud content [%]	1.6	≤3
Limestone filler	Density [g/cm^3^]	2.71	/
	Moisture content [%]	0.23	≤1
	Hydrophilic coefficient [%]	0.67	≤1

**Table 3:** Failure load and maximum deformation of different types of PACM.

	PC-10	PC-16	PC25
Failure load	3.96 kN	2.67 kN	3.23 kN
Maximum deformation	1.74 mm	1.66 mm	2.89 mm

**Table 4:** Dissipated angle of PACM under different gradation.

Type	Stress Level	Dissipated Angle (°)								
		2 Hz	SD	N	5Hz	SD	N	10 Hz	SD	N
PC-10	0.3	24.5	1.23	21,738	23.1	1.16	54,019	21.6	1.08	90,227
	0.5	29.3	1.47	14,252	28.5	1.43	43,294	28.2	1.41	70,970
	0.7	34.7	1.74	10,001	31.9	1.60	36,131	30.4	1.52	42,835
PC-16	0.3	26.8	1.34	13,172	25.2	1.26	52,008	23.5	1.18	83,743
	0.5	31.2	1.56	10,235	29.6	1.48	42,756	28.8	1.44	61,645
	0.7	37.5	1.88	4844	35.3	1.77	23,882	34.2	1.71	39,249
PC-25	0.3	35.1	1.76	8317	31.6	1.58	46,141	27.4	1.37	75,664
	0.5	41.7	2.09	3221	36.2	1.81	35,919	33.1	1.66	52,078
	0.7	48.3	2.42	1445	43.9	2.20	20,103	39.5	1.98	36,404

**Table 5:** Cumulative dissipated energy of PACM under different gradation.

Type	Stress Level	Cumulative Dissipated Energy (MJ/m^3^)					
		2 Hz	SD	5 Hz	SD	10 Hz	SD
PC-10	0.3	68.388	3.42	153.261	6.13	184.965	9.25
	0.5	59.858	2.99	124.244	6.21	178.135	8.91
	0.7	43.204	2.16	118.149	5.91	112.228	5.61
PC-16	0.3	40.735	2.04	147.936	7.40	181.722	9.09
	0.5	38.725	1.94	131.580	6.58	146.099	7.30
	0.7	20.781	1.04	84.781	4.24	118.140	5.91
PC-25	0.3	25.367	1.27	125.357	6.27	158.138	7.91
	0.5	13.271	0.66	115.814	5.79	125.508	6.28
	0.7	6.387	0.32	70.159	3.51	113.945	5.70

**Table 6:** Fitting parameters of prediction model of fatigue life of PACM with different grading types.

Grading Types	A	B	R^2^
PC-10	1.6146	−1.095	0.9714
PC-16	2.1717	−1.072	0.9826
PC-25	0.0864	−1.515	0.9944

## Data Availability

The original contributions presented in the study are included in the article, further inquiries can be directed to the corresponding author.

## References

[1] Zofka A., Błażejowski K., Ostrowski P. (2021). Fatigue performance of asphalt pavements with highly polymer-modified asphalt binders. Road Mater. Pavement Des..

[2] Dan H.C., Lu B., Li M. (2024). Evaluation of asphalt pavement texture using multiview stereo reconstruction based on deep learning. Constr. Build. Mater..

[3] Hu M.J., Lyu L., Pahlavan F., Han P., Sun D., Fini E.H. (2025). Toward sustainable non-emitting asphalts: Understanding diffusion-adsorption mechanisms of hazardous organic compounds. Adv. Sustain. Syst..

[4] Yang Q.L., Zhu Q., Xu R., Hao L., Lu G., Wang D. (2025). Synergism and Mechanisms of Aging Resistance in the Use of Graphene and Carbon Nanotubes in Bitumen Composites. Langmuir.

[5] Saha D.C., Mandal J.N. (2020). Performance of reclaimed asphalt pavement reinforced with Bamboo geogrid and Bamboo geocell. Int. J. Pavement Eng..

[6] Fu C.L., Liu K., Liu Q., Xu P., Dai D., Tong J. (2023). Exploring directional energy conversion behavior of electromagnetic-based multifunctional asphalt pavement. Energy.

[7] Hu Y.P., Sreeram A., Al-Tabbaa A., Airey G.D. (2025). Physicochemical compatibility assessment of bio-additives and bitumen using solubility science-based approaches. Fuel.

[8] Liu K., Zhang Y., Wang F., Da Y., Zhang H., Pang H. (2025). Investigation on the healing effects of microwave heating in eco-friendly pavement using e-waste. Mater. Struct..

[9] Xiong K., Zhang J., He Y., Li J., Zhang M., Li R., Pei J., Li Y., Lyu L. (2024). Introducing the mineral powder to strengthen polyurethane grouting materials for crack repair of asphalt pavements. Constr. Build. Mater..

[10] Vennapusa P.K.R., White D.J. (2015). Field assessment of a jointed concrete pavement foundation treated with injected polyurethane expandable foam. Int. J. Pavement Eng..

[11] Cui C., Guo C., Lu Q., Wang F., Fang H. (2021). Fatigue performance of concrete–polyurethane composite materials under compression. J. Transp. Eng. Part B Pavements.

[12] Liu W., Zhang S., Li Y., Ye X. (2023). The expansion and mechanical property-based cavity expansion model for polyurethane grouting underneath the airport pavement. Transp. Geotech..

[13] Wang F.M. (2018). Practice of non-water-reacting polymer grouting treatment to seepage. J. Hydroelectr. Eng..

[14] Gao X., Wei Y., Wang F.M., Zhong Y.H. (2017). Fatigue performance and microstructure evolution of polyurethane grouting materials under cyclic compression loading. Acta Mater. Compos. Sin..

[15] Wei Y. (2017). Microstructure and fatigue performance of polyurethane grout materials under compression. J. Mater. Civ. Eng..

[16] Liu K., Tong J., Huang M., Wang F., Pang H. (2022). Model and experimental studies on the effects of load characteristics and polyurethane densities on fatigue damage of rigid polyurethane grouting materials. Constr. Build. Mater..

[17] Haruna S.I., Ibrahim Y.E., Han Z., Farouk A.I.B. (2023). Flexural response of concrete specimen retrofitted with pu grout material: Experimental and numerical modeling. Polymers.

[18] Zhou K., Liang H., Huang F., Cheng Z. (2024). Evaluation for anti-cracking performance of polyurethane grout based on overlay test. Front. Built Environ..

[19] Opland W.H., Barnhart V.T. (1995). Evaluation of the URETEK Method for Pavement Undersealing.

[20] Crawley A.B. (1996). Evaluation of the Uretek Method for Pavement Undersealing and Faulting Correction.

[21] Zhong Y.H., Xu S., Chi J., Zhang B., Shen G., Yang Z., Chen H., Wang B. (2023). Experimental study on the interface bonding characteristic of polyurethane and pavement materials. Constr. Build. Mater..

[22] Ran M.P., Zhou X.X., Yan Y., Jiang R.Q., Zhou X.L. (2023). Grouting mechanism of polyurethane composite materials in asphalt pavement subsidence. Materials.

[23] Wu H., Shu Y., Liu Y. (2017). Engineering performance of polyurethane bonded aggregates. Mater. Sci..

[24] Zhou J.L., Zheng M., Wang Q., Yang J., Lin T. (2016). Flexural fatigue behavior of polymer-modified pervious concrete with single sized aggregates. Constr. Build. Mater..

[25] Yeon K.S., Choi Y.S., Kim K.K., Yeon J.H. (2017). Flexural fatigue life analysis of unsaturated polyester-methyl methacrylate polymer concrete. Constr. Build. Mater..

[26] Ghassemi P., Rajabi H., Toufigh V. (2020). Fatigue performance of polymer and ordinary cement concrete under corrosive conditions: A comparative study. Eng. Fail. Anal..

[27] Xu S.F., Xu M., Zhang Y., Guo Y., Peng G., Xu Y. (2020). An indoor laboratory simulation and evaluation on the aging resistance of polyether polyurethane concrete for bridge deck pavement. Front. Mater..

[28] Xu S.F., Xu M., Fang C., Liu H., Ren X., Han B. (2022). Laboratory investigation on traffic opening timing of polyether polyurethane concrete. J. Test. Eval..

[29] Jia Z., Jia D., Sun Q., Wang Y., Ding H. (2021). Preparation and mechanical-fatigue properties of elastic polyurethane concrete composites. Materials.

[30] (2005). Technical Specifications for Construction of Highway Asphalt Pavements.

[31] (2012). Standard Test Methods of Bitumen and Bituminous Mixtures for Highway Engineering.

[32] Sun Y., Fang C., Wang J., Ma Z., Ye Y. (2018). Energy-based approach to predict fatigue life of asphalt mixture using three-point bending fatigue test. Materials.

[33] Shen S.H., Airey G.D., Carpenter S.H., Huang H. (2006). A dissipated energy approach to fatigue evaluation. Road Mater. Pavement Des..

[34] Ghuzlan K.A., Carpenter S.H. (2000). Energy-Derived, damage failure criterion for fatigue testing. J. Transp. Res. Rec..

[35] Carpenter S.H., Ghuzlan K.A., Shen S. (2003). Fatigue endurance limit for highway and airport pavements. J. Transp. Res. Rec..

[36] Wu J., He Y., Yu Z. (2020). Failure mechanism of rigid polyurethane foam under high temperature vibration condition by experimental and finite element method. J. Appl. Polym. Sci..

[37] Lou K.K., Wu X., Xiao P., Zhang C. (2021). Investigation on fatigue performance of asphalt mixture reinforced by basalt fiber. Materials.

